# Seed-Mediated Electroless Deposition of Gold Nanoparticles for Highly Uniform and Efficient SERS Enhancement

**DOI:** 10.3390/nano9020185

**Published:** 2019-02-01

**Authors:** Junqi Tang, Quanhong Ou, Haichun Zhou, Limin Qi, Shiqing Man

**Affiliations:** 1 Yunnan Key laboratory of Optoelectronic Information Technology, College of Physics and Electronic Information, Yunnan Normal University, Kunming 650500, Yunnan, China; tjunqi@163.com (J.T.); ouquanhong@163.com (Q.O.); haichunzhou@shu.edu.cn (H.Z.);; 2 Beijing National Laboratory for Molecular Sciences, College of Chemistry, Peking University, Beijing 100871, China; liminqi@pku.edu.cn

**Keywords:** electroless deposition, nanoparticles, nanofilm, SERS, in situ chemical reduction

## Abstract

A seed-mediated electroless deposition (SMED) approach for fabrication of large-area and uniform gold nanoparticle films as efficient and reproducible as surface-enhanced Raman scattering (SERS) substrates was presented. This approach involved a seeding pretreatment procedure and a subsequent growth step. The former referred to activation of polylysine-coated glass slides in gold seed solution, and the latter required a careful control of the reactant concentration and reaction time. With the aid of gold seeds and appropriate reaction conditions, a large-area and uniform nanofilm with evenly distributed gold nanoparticles (Au NPs) was formed on the surface of the substrates after adding a mixed solution containing ascorbic acid and trisodium citrate. The morphology of the Au nanofilm was examined by scanning electron microscopy. The size evolution of Au NPs on the surface of the substrates was analyzed in detail. The nanofilm substrate was prepared by reaction conditions of the seeded activation process: 10 mL ascorbic acid and trisodium citrate mixture and 30 min of soaking time, which exhibited an excellent uniformity and reproducibility of SERS enhancement with relative standard deviation (RSD) values of less than 8% (particularly, a RSD value of 3% can be reached for the optimized measurement). Compared to the common electroless deposition, the seed-mediated electroless deposition possessed inherent advantages in controllability, reproducibility, and economic benefit.

## 1. Introduction

The term “electroless plating” (EP or ELP) is referred to as the spontaneous reduction or in situ reduction of metal ions to the metallic state in the absence of an external source of electric current, which was firstly coined by Brenner and Riddell and is also generally referred to as “in-situ chemical reduction”, “chemical deposition” or “electroless deposition” [1,2,3,4]. This method has been widely used for the preparation of metal films on various substrates, such as metal on metal, metal on semiconductor, and metal on insulator [1]. For instance, Tsuneyoshi and Ono [3] fabricated Ni–P alloy film-coated microcapsules with a core-shell structure via ELP. Milazzo et al. [5] investigated electroless deposited Au NPs on a 3C–SiC substrate. In another study, Yadav et al. [6] exploited an electroless gold plating process to metallize electrospun polyacrylonitrile (PAN) nanofibers based on the non-cyanide gold plating bath. The gold-coated PAN nanofibers had good electrical conductivity and improved hydrophobic characteristics. As for other common substrates, such as glass slide, Si wafer, and mica plate, there were many reports about electroless deposition of metal nanoparticles on these substrates [7,8,9,10,11]. 

In contrast to other coating techniques, such as sputtering, chemical vapor deposition (CVD), ion beam assisted deposition, pulsed vacuum arc deposition (PVAD) [12,13,14], electroplating, electrochemical or electrophoresis deposition [15,16], EP required neither complicated, expensive equipment nor sophisticated operation. This method has been developed to fabricate large-area, uniform and reproducible substrates for surface-enhanced Raman scattering (SERS) [17,18,19,20]. 

Surface-enhanced Raman scattering spectroscopy technology is a powerful analytical tool for real-time and ultra-high sensitive detection of the intended analyte in numerous fields such as food safety, laboratory medicine, chemical sensing, and environment monitoring. The SERS technique can achieve single molecule level detection with nearly 14 orders of magnitude Raman signal enhancement, which has been attracting a lot of attention [21,22,23,24,25]. For example, Xu et al. [26] created a highly-uniform and ordered SERS substrate on hydrophobic Si nanopillar array by droplet-confined electroless deposition (DCED) method. This substrate exhibited an excellent reproducibility in SERS measurements [26]. Similarly, a space confined method was developed to fabricate SERS-active substrates with silver nanoparticles (AgNPs) on a Si nanopillars array. The AgNPs were uniformly distributed on a template of Si nanopillars array during the electroless metal deposition (EMD) process. This substrate also showed good SERS enhancement performance [27]. Cheng and Yang [7] developed an electroless deposition method for AgNPs on glass substrates for SERS measurements. To improve sensitivity and variability of SERS signals, a seed-mediated growth method was employed in their work. Actually, the “seed-mediated” growth method was adopted to prepare metal nanoparticles with different sizes and shapes for a long period of time, such as in aqueous media [28,29,30] and on different substrate surfaces [31,32]. Wang et al. [33] proposed an electrochemical seed-mediated method for deposition of gold nanoparticles (Au NPs) on ITO electrode. The gold nanoparticle array with controlled particle size and density had rich “hot spots”, which could produce high performance of SERS. Philip et al. [34] has synthesized a kind of large-sized Au NPs via a seed-mediated growth method and fabricated SERS substrates based on electrostatic interaction between positively charged polyethylenimine-capped Au NPs and negatively charged surfaces of silicon oxide supports. In order to develop an effective and reliable SERS substrate, the fabrication of uniform and ordered nanoparticles films with rich hot spots is of great significance. Additionally, it has been widely agreed that an ideal SERS substrate for sensing applications in practice should induce a high signal enhancement, generate a reproducible and uniform response, have a stable half-life, and be easily synthesizable [35]. However, the challenges in the facile, cost-effective, and versatile fabrication of large-area, reproducible, and uniform SERS substrates still remain.

In the present study, we proposed a modified electroless deposition method, named seed-mediated electroless deposition (SMED), to fabricate uniform, large-area, and efficient SERS-active substrates based on commercial polylysine-coated glass slides. A key point of SMED method was the use of gold seed nanoparticles to activate surface groups of the polylysine-coated glass slides substrate. Growth of Au nanoparticle films was carried out by dropwise adding a mixture solution of ascorbic acid (AA) and trisodium citrate (Na_3_Cit) to the HAuCl_4_ reaction solution. The AA served as reducing agent while the Na3Cit served as coating agent. The gold atom was firstly reduced in electroless gold solutions. Then, Au atom and Au NPs were homogeneously and orderly immobilized on the surface of the polylysine-coated glass slide. The SERS performance of the as-prepared large-area and uniform Au nanoparticles film was systematically investigated using crystal violet (CV) as the Raman probe molecules. With the optimized reactant concentration and deposition time, the SERS substrate exhibited excellent uniformity and reproducibility with relative standard deviation (RSD) values of less than 8%. Additionally, the procedure was quite facile, economical, and flexible. These characteristics endow SMED method with the many advantages for preparing large-area and uniform nanoparticle films for routine SERS applications.

## 2. Materials and Methods

### 2.1. Materials

Gold chloride trihydrate (HAuCl_4_·3H_2_O, 99.9%), sodium borohydride (NaBH_4_, 98%), sodium citrate dihydrate (trisodium citrate or Na_3_Cit, 99%), ascorbic acid (AA, 99%), and sodium hydroxide (NaOH, 99.9%) were purchased from Shanghai Aladdin Chemical Reagent Co., Ltd. (Shanghai, China). Polylysine-coated glass slides (Adhesion Microscope Slide, 75 mm × 25 mm × 1.1 mm) and crystal violet (CV) were purchased from Yifan equipment company (Nantong, China) and Tianjin Kemiou Chemical Reagent Co., Ltd. (Tjianjin, China), respectively. All chemicals were of analytical grade and used without any further purification. Ultrapure water (Resistivity > 18.25 MΩ·cm) was used for all solution preparation and throughout the experiments. The polylysine-coated glass slides were snapped to pieces (about 1.5 × 2.5 cm^2^) with the help of a glasscutter and subsequently soaked in ultrapure water for further surface modification.

### 2.2. Instrumentation

A Shimadzu UV-2600 was used to characterize ultraviolet–visible (UV-vis) spectra of gold seeds and the localized surface plasmon resonance (LSPR) of the obtained SERS substrates. All spectra of the substrates were measured in the range from 300 to 900 nm with a resolution of 0.5 nm. 

Transmission electron microscopy (TEM) images were measured with a JEOL JEM-2100 electron microscope at an accelerated voltage of 200 kV. The morphology of the large-area and uniform films was characterized by scanning electron microscopy (SEM, Nova NanoSEM 250 instrument, FEI Company, Hillsboro, OR, USA) with an accelerating voltage of 30 kV.

A confocal Raman microscope was used to perform Raman experiments. This equipment mainly comprised a 532 nm green solid-state laser (Cobolt Samba 532 nm, Cobolt AB, Solna, Sweden), a Shamrock SR-500i-B2 spectrograph (focal length 500 mm, 600 lines/mm grating, a spectral resolution of approximately 0.13 nm corresponding to ~4 cm^−1^), and a charge-coupled device (CCD) camera fitted with a back-illuminated deep-depletion CDD-chip (Andor iDus 416, DU416A LDC-DD, Andor Technology Ltd., Belfast, UK). The spectrograph was coupled to a microscope (Leica DM 2700M, Leica Microsystems Wetzlar GmbH). A 50× (NA = 0.5) objective lens was used to focus the laser beam onto the sample surface and the scattered signal was collected by the thermo-electrically cooled CCD camera (Andor Technology Ltd., Belfast, UK). The camera was cooled to −70 °C in order to decrease thermal noise. An edge filter was employed to block stray light. Andor SOLIS software (Andor Technology) was used for spectral data acquisition. Wavenumbers ranging from 400 cm^−1^ to 2000 cm^−1^ were examined here, which covered most of the characteristic Raman lines of the investigated analytes. A slit width of 100 µm was used. The laser power on the sample position was about 10 mW. The spectra were recorded with an integration time of 5 s and the number of accumulations was 2 for all measurements.

### 2.3. Procedures

#### 2.3.1. Preparation of Gold Seed Solutions

In this study, two kinds of gold seeds with different sizes were prepared according to a modified NaBH_4_ reduction method [36]. The first one was that 100 μL of 0.034 M Na3Cit solution was added into a 10 mL of 0.025 mM HAuCl_4_ solution, which was placed in an ice bath. Then, 500 μL of newly prepared ice-cold 0.01 M NaBH_4_ solution was quickly added into the mixture of HAuCl_4_-citrate and the color of solution was immediately changed to light purple. The colloidal solution was marked as seed I. For the preparation of the other seed solution, 25 μL of 0.1 M Na_3_Cit solution was added into 10 mL of ice cold 0.25 mM HAuCl_4_ solution. Then, 300 μL of newly-prepared ice-cold 0.1 M NaBH_4_ solution was quickly added. This seed solution was marked as seed II. The two seed solutions were continuously stirred in an ice water bath until the completion of the reaction. Subsequently, the polylysine-coated glass slides were immersed in the seed I or the seed II to modify the surface properties. Sodium borohydride was used as the strong reducing agent to form gold nanoparticles. Adding lots of citrate was to help stabilize gold nanoparticles and also to prevent gold nanoparticles from growing and aggregating.

#### 2.3.2. Fabrication of Large-Area and Uniform Substrate Films by SMED

**Figure 1:**
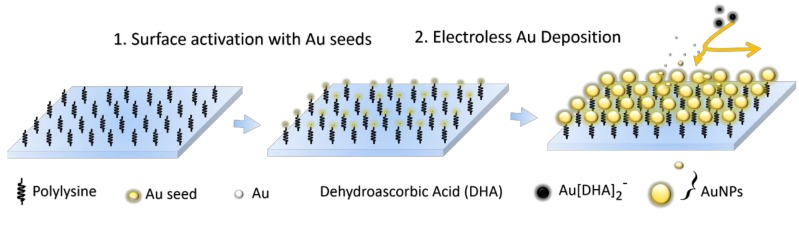
Schematic diagram of fabrication of Au nanoparticle films as uniform SERS-active substrates by SMED method.

The preparation of large-area and uniform substrates was based on a seed-mediated electroless deposition (SMED) method. In all cases, the polylysine-coated glass slides were soaked in a gold seed solution and placed in a fridge at 6 °C overnight (about 12 h) to activate the surface groups. The gold seed nanoparticles can be preferentially attached to the polylysine-coated glass slide surface because of the rich amine end groups. Unfixed gold seeds were washed away with copious amounts of ultrapure water. After the surface of the glass slides was activated and modified with small gold seed nanoparticles, the polylysine-coated glass slides were dipped into a 10 mL of 1 mM HAuCl_4_ solution. Subsequently, 10 mL of ascorbic acid and trisodium–citrate mixture solution (0.05% and 0.025%, *w*/*v*) was dropwise added to the HAuCl_4_ solution by a micropump with a speed of 20 mL/h. After adding ascorbic acid as a reducing agent, the chloroauric acid anion (AuCl_4_^−^) was reduced to gold atom. Simultaneously, the gold atoms were agglomerative and self-assembled into a large-area and uniform gold nanoparticle films on the glass substrate. After different immersion times, the size of Au NPs on the substrate and the gap between them can be easily controlled. Finally, the prepared substrates were thoroughly rinsed with ultrapure water to remove remaining reactants and most of physically adsorbed Au NPs. The detailed process was schematized in Figure 1.

In the absence of seed solution, the polylysine-coated glass slides were firstly soaked in 10 mL of 1 mM aqueous solution of HAuCl_4_. Then, a 10 mL of the mixture containing trisodium citrate (0.025%) and ascorbic acid (0.05%) was dropwise added into this reaction solution with a dropping speed of 20 mL/h.

#### 2.3.3. SERS Spectroscopy of CV on SERS-Active Au Nanoparticles Film

For SERS measurements, the prepared SERS-active Au nanoparticles film substrates were incubated in 1 × 10^−6^ M CV aqueous solutions for 15 min. The substrates were then rinsed thoroughly with ultrapure water to remove the unbound CV molecules (CVs). Finally, SERS measurements were executed after the substrates were dried at room temperature for 30 min. 

## 3. Results and Discussion

The polylysine-coated glass slide can provide superior cell adhesion capacity. This feature of glass slide has been commonly used as supports for protein, bacteria, cell, and DNA attachment. In the field of SERS, there have been almost no reports on preparation of large-area and uniform SERS-active substrates using the polylysine-coated glass slide. Until now, some researchers have employed the polylysine-coating glass slide to improve the uniform distribution of the adsorbed nanoparticles [37,38,39]. So, it inspired us to fabricate a simple, inexpensive, large-area, and uniform SERS substrate without laborious modification processes. All of prepared substrates with corresponding different reaction conditions such as gold seed, reactant concentration, and reaction time were listed in Table 1.

### 3.1. Effect of Gold Seed

The absorption peaks of these colloidal gold (Au) seed solutions were at 503 (blue curve) and 512 nm (red curve) which are shown in Figure S1. It is well known that the LSPR absorption peak red-shifts as the size of nanoparticles increased. According to the previous theoretical calculation [40] and experimental results [41], the average sizes of the two types of gold seeds were about 2 nm and 5 nm, respectively. The different sizes of gold seeds would affect the surface modification of the glass substrate.

**Figure 2:**
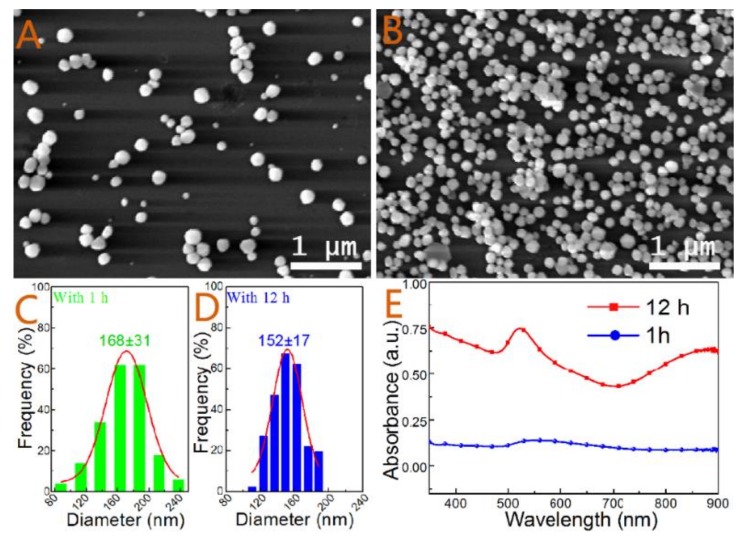
SEM images of the substrates with immobilized Au NPs without activation process at different immersion times: (**A**) 1 h and (**B**) 12 h. (**C**) and (**D**) Particle size distributions of Au NPs in (**A**,**B**,**E**) UV-Vis spectra of the substrates with immobilized Au NPs prepared with different immersion times

Although many studies have shown that the seeded activation of substrates, such as Si wafer or glass slide, was helpful for preparation of silver mirror-like metallic coatings, these studies were rarely concerned about the controllability and uniformity of the whole SERS substrate. In order to avoid formation of smooth coatings or troublesome post-synthetic treatments and to better understand the role of the seed, the seeded activation process was carefully examined. Figure 2 showed the SEM images, particle size distributions, and UV-Vis spectra of the prepared substrates with different reaction times. There were only a few sparse Au NPs settled on the substrate after a short immersing time of 1 h (Figure 2A). The average size and standard deviation of the random Au NPs were 168 ± 31 nm based on more than 100 nanoparticles (Figure 2C). The high relative standard deviation (RSD ~18.5%) and sparse dispersion of Au NPs were unsuitable for uniform and efficient SERS measurements. After extending the immersion time to 12 h, the density of Au NPs was significantly increased under the same electroless gold solution (Figure 2B). However, it was interesting that the mean and standard deviation of the immobilized Au NPs were only 152 ± 17 nm (Figure 2D). The decreased grain size and relative standard deviation (RSD ~11.2%) of adsorbed Au NPs suggested that there could be a self-adjustment and reshaping in the long secondary process. Figure 2E showed the UV-Vis spectra of the two substrates. As extending the immersion time, the LSPR was obviously enhanced at 540 nm. There was an intensive peak at about 880 nm because of particles aggregation. Based on these results, it can be inferred that the increased density of Au NPs might be associated with an electrostatic interaction and a secondary growth process. In the HAuCl_4_ solution, the abundant NH_3_^+^ groups due to protonation would form at the surface of the substrate. When the solution of trisodium citrate and ascorbic acid were added, Au^3+^ ions were firstly reduced to Au^1+^ ions, followed by the form of atoms (Au^0^) and small Au NPs. The small Au NPs were able to accumulate and assemble to form larger Au NPs in solution. Meanwhile, the Au NPs were negatively charged because of adsorption of AuCl_4_^−^ and AuCl_2_^−^ ions [42]. The positively charged surface of the polylysine-coated glass slides can effectively adsorb a great number of negatively charged small Au NPs owing to electrostatic adsorption. However, though the density of immobilized Au NPs was increased with increasing the soaking time, the adsorbed Au NPs did not grow to larger sizes. But a more uniformed size distribution appeared in the secondary growth process. The result indicated that there was other reaction for this phenomenon. For all this, a closed-packed arrangement and ordered Au NPs film became more difficult to generate because of the co-action of electrostatic repulsion and steric hindrance of larger Au NPs.

**Figure 3:**
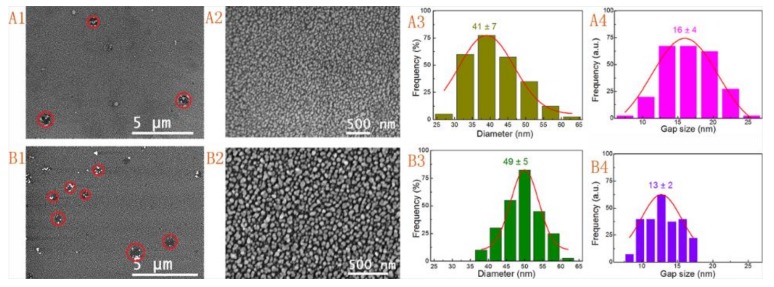
SEM images (**A1**,**A2**,**B1**,**B2**), size distributions (**A3**,**B3**), and gap size distributions (**A4**,**B4**) of Au nanoparticle films formed by utilizing seed I activation and with an immersion time of 30 min (**A1**–**A4**) and seed II activation combined with an immersion time of 12 h (**B1**–**B4**).

To further study the effect of Au seeds on the morphology of the substrates, two types of Au seeds with different sizes were employed to activate the surface of the polylysine-coated glass slides. In the presence of gold seeds, the activated substrates were soaked in electroless gold solutions with at least 30 min after adding 10 mL of reaction solution. Then, large-area and uniform gold nanoparticle films were formed with this SMED method (Figure 3). The observed SEM images showed that both substrates were covered with evenly distributed and closed-packed nanoparticles after treating with seed I and seed II. Compared to the Au deposition without seeding activation process (Figure 2), these results suggested that the Au seed activation was an essential procedure for fabricating large-area and uniform Au nanoparticle films (Figure 3A1,A2,B1,B2). The average size of Au NPs and the mean interparticle gaps for the substrate prepared by employing seed I activation and with immersion time of 30 min were 41 ± 7 nm and 16 ± 4 nm, respectively (Figure 3A3,A4). Similarly, the values for another substrate prepared by employing seed II activation combined with a soaking period of 12 h were 49 ± 5 nm and 13 ± 2 nm, respectively (Figure 3B3,B4). The larger Au NPs were attributed to the larger seeds and the smaller RSD values were due to the different immersion times. The fabricated substrates with different sizes and immersion times showed no significant difference in the morphology and structure, which indicated that the sizes of the seed had no discernible effect on fabrication of substrates. But the immersion time seemingly influenced the mono-dispersity of the Au NPs and the mean size of interparticle gaps. Though Au NPs were distributed fairly uniformly on the whole surface; however, there were some collection regions of Au NPs (or called defect regions which were marked with red circles in Figure 3A1,B1). These defective regions were not completely covered with Au NPs both in two kinds of substrates. We proposed that such defects could derive from the processing of the commercial glass slide.

### 3.2. Effect of Reactant Concentration

**Figure 4:**
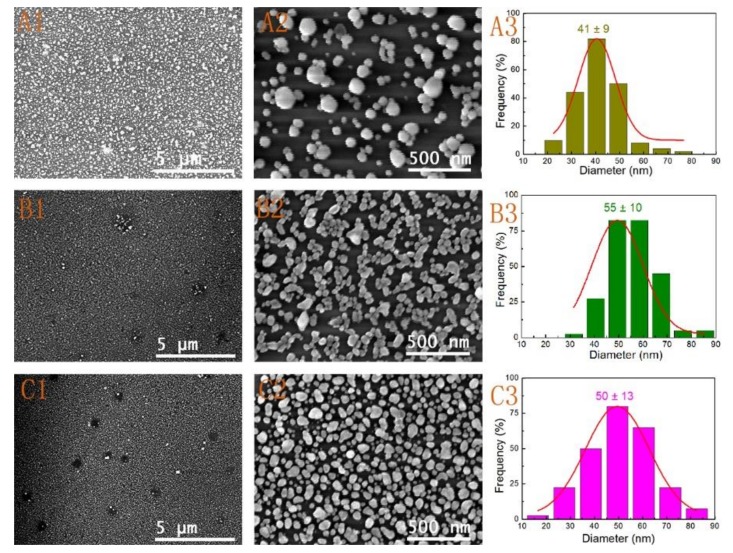
SEM images (**A1**,**A2**,**B1**,**B2**,**C1**,**C2**) and size distributions (**A3**,**B3**,**C3**) of Au nanoparticle films formed by adding different amounts of reaction mixture after the substrates were activated with seed I: (**A1**–**A3**) 1 mL, (**B1**–**B3**) 2 mL, (**C1**–**C3**) 5 mL (All of soaking time was fixed at 12 h).

The above analysis suggested that the seeding pretreatment procedure was the foundation of fabricating large-area and uniform Au nanoparticle films. However, the different parameters during the growth process are also important factors to influence the morphology and structure evolution of these substrates. These parameters mainly include the reactant concentration and reaction time. Based on the results in Figure 3A1–A4, we firstly evaluated the effect of the amount of reaction mixture after the substrates activated were with seed I. The amount of reaction mixture was decreased from 10 mL to 1 mL, while the soaking time was all fixed at 12 h. Figure 4 shows the SEM images and size distributions, and UV-Vis spectra (Figure S2) of the Au nanoparticle films with different amounts of trisodium citrate and ascorbic acid reaction mixture. The substrate shown in Figure 4A1,A2 was obtained with the addition of only 1 mL of reaction mixture to the HAuCl_4_ solution. The substrate was soaked in electroless gold solution for 12 h. The size of the formed Au NPs was about 41 ± 9 nm without consideration of some oversized Au NPs, and the entire surface of the substrate was not completely covered (Figure 4A1–A3). The non-uniformity and low-density of the Au NPs were due to the too low amount of reaction solution. As the mixed solution was increased to 2 mL, the stacking density of the nanoparticles was obviously increased (Figure 4B1,B2), and the size of the Au NPs grew to 55 ± 10 nm (Figure 4B3). However, there were still a lot of large void areas and loose nanogaps. These results suggested that it was hard to form large-area and uniform nanofilm only by expanding immersion time at the low reactant concentration. When the amount of reaction mixture over 5 mL, Au NPs were uniformly distributed on the whole substrate. Figure 4C1,C2 represented the low- and high-magnification SEM images of the substrate obtained by adding 5 mL of reaction mixture. The uniformity and stacking density of Au NPs obviously improved. Compared to the large-area and uniform substrate prepared by utilizing seed I activation and with an immersion time of 30 min (Figure 3A1–A2), the results suggested that the substrate with a favorable homogeneity and compactness required a higher reactant concentration and an appropriate immersion time. According to statistics, the sizes of Au NPs on the substrate prepared by adding 5 mL of reaction mixture were 50 ± 13 nm (Figure 4C3). Additionally, it can be seen that there were scarcely any larger Au NPs gathered around the defect areas after adding only 5 mL of reaction mixture (Figure 4C2). Due to a short period of immersion time (30 min), there were also hardly any Au NP aggregations in the defect areas after adding 10 mL of mixed solution (Figure 3A1–A2). This result clearly indicated that both the amount of reaction mixture and the immersing time were of importance for preparing large-area, uniform, and ordered SERS-active substrates. Moreover, all of Au nanoparticle films exhibited the characteristic of LSPR (Figure S2). As expected, the strength of the spectrum band increased with increasing the surface coverage and stacking density. During the course of electroless deposition with increasing the reactant concentration, the characteristic absorption peak of the Au NPs at 520 nm was red-shifted. A shoulder peak at about 750 nm appeared and then slightly shifted to shorter wavelength (Figure S2). Finally, the two LSPR peaks were difficult to distinguish due to the formation of a close-packed film. It was worth noting that the LSPR extinction efficiency strongly depended on the particle size, shape, composition, and the dielectric constant of the surrounding medium (including the refractive index of the surrounding solvent and the chemical surface functionalization of nanoparticle). The SPR feature of the substrates can be ascribed to the synergetic effects of the single NP plasmon oscillation and the interparticle plasmon coupling oscillation [43,44].

### 3.3. Effect of Reaction Time

**Figure 5:**
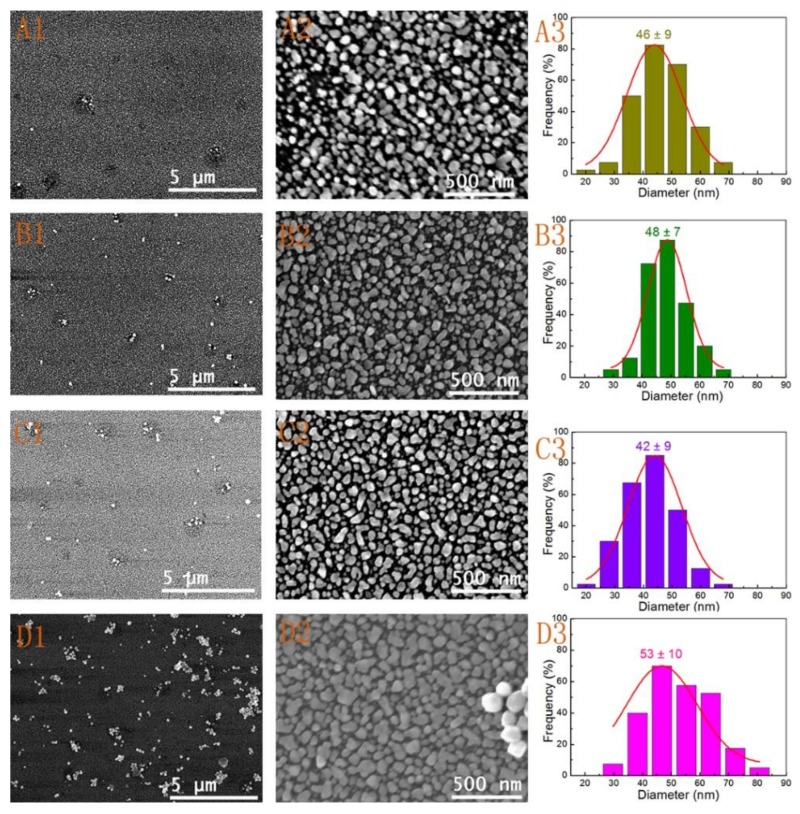
SEM images (**A1**,**A2**,**B1**,**B2**,**C1**,**C2**,**D1**,**D2**) and size distributions (**A3**,**B3**,**C3**,**D3**) of the obtained Au nanoparticle films after activating with seed II and soaking with different reaction times: (**A1**–**A3**) 15 min, (**B1**–**B3**) 30 min, (**C1**–**C3**) 5 h, (**D1**–**D3**) 20 h. The fabricated substrate was activated by seeds II and combined with immersion time of 12 h showed in Figure 4B1–B3.

As previously mentioned, the grain sizes, coverage, and uniformity of the deposition substrates were also depended on the immersion time. Nevertheless, an extended immersion time would cause the physical adsorption and aggregation of larger Au NPs in the defect areas. Thus, we discussed the effect of immersion time on Au nanoparticle films in detail. When the reaction mixture was added, the electroless deposition process was triggered and Au NPs were rapidly formed on the polylysine-coated glass slide substrate. It was shown in Figure 5 that the different stages of the deposition had an obvious impact on the morphology of the substrates activated with seed II. Firstly, Figure 5A1,A2) showed the SEM images of the Au NPs film formed by adding a half amount of mixed solution (about 5 mL) with time of 15 min. It can be seen that the Au NPs film was formed rapidly with only a few defect points. The structure was similar with the result shown in Figure 4C1, which was obtained by activation with seed I after a longer immersion time. When adding the reaction solution was finished within 30 min, the defect regions were decreased and the surface grain size increased slightly from 46 ± 9 to 48 ± 7 nm (Figure 5A3,B1–B3). Some Au NPs started to gather around these defect points (defect regions) as the immersion time increased to over 5 h (Figure 3B1 and Figure 5C1,D1). The images in Figure 5C1,D1 represented the substrates obtained by immersing in the electroless solution for 5 h and 20 h, respectively. From Figure 5C2, we can clearly see that the substrate was covered with uniform and homogeneous gold nanoparticles. But the size of the Au NPs was decreased to 42 ± 9 nm (Figure 5C3). If the substrate was soaked in the solution for over 20 h (Figure 5D1), a lot of large Au NPs were deposited on the whole gold nanoparticle film. The physical adsorption of gathered Au NPs would have a significant effect on the uniformity of the film. According to the image shown in Figure 5D3, the size of the Au NPs grew to 53 ± 10 nm. However, some Au NPs began to form continuous island structures. More intriguing was that the size of Au NPs undergoes the first increase, then decrease, and again increase for the formation of island structure. This phenomenon of size evolution of Au NPs was observed for both extending immersion time and increasing added mixture solution. This also indicated that prolonging soaking time did not always improve the uniformity of Au nanoparticles film. The Au NPs film might grow to be a continuous nano-island structure and some Au NPs would deposit on the surface of the nanofilm. Thus, the immersion time needs to be controlled carefully. From all of these results, a mechanism was proposed to explain the size evolution of Au NPs. It was well known that [Au^III^Cl_4_]^−^ could be readily reduced to [Au^I^Cl_4_]^-^ by ascorbic acid (AA) (Equation (1)). The [Au^I^Cl_4_]^−^ was a meta-stable species, which would be further reduced to Au metal and AA was oxidized to DHA. The atomic gold quickly became Au NPs in solution or adsorbed on the surface of the substrate (Equations (2) and (3) [45,46]. The small Au NPs gradually grew by increasing the added the amount of mixture or prolonging the immersion time at this stage. It was mainly reflected in Figure 4A1,B1) and Figure 5A1,B1. As the substrate was soaked in the electroless solution in an extended period, a slow disproportionation reaction occurred (Equation (4)), which can explain why the size of Au NPs decreased, as shown in Figure 2, Figure 4B1,C1 and Figure 5B1,C1. At last, the size of Au NPs was increased again for the reduction of [Au^I^Cl_4_]^−^ in the following immersion time. Thus, a careful control of the reactant concentration and immersion time would enable the preparation of large-area and uniform gold nanoparticle films.

(1) $${\left[{\mathrm{Au}}^{\mathrm{III}}{\mathrm{Cl}}_{4}\right]}^{-}+2C_{6}H_{8}O_{6}={\left[Au^{I}Cl_{2}\right]}^{-}+2Cl^{-}+2H^{+}+2C_{6}H_{6}O_{6},$$

(2) $${\left[{\mathrm{Au}}^{I}{\mathrm{Cl}}_{2}\right]}^{-}+e^{-}=Au^{0}+2Cl^{-}$$

(3) $$n\left({\mathrm{Au}}^{0}\right)\overset{\;\;\;\;\mathrm{fast}\;\;\;\;}{\to }{\left(Au\right)}_{n}$$

(4) $$2\;Au^{0}+{\left[Au^{\mathrm{III}}Cl_{4}\right]}^{-}+2\;Cl^{-}\overset{}{\Leftrightarrow }3{\left[{\mathrm{Au}}^{I}Cl_{2}\right]}^{-}$$

### 3.4. SERS Enhancement Performance

Now it is widely recognized that the SERS’ effect primarily originates from electromagnetic (EM) enhancement and chemical (CHEM) enhancement effects [47]. Giant EM enhancement mainly arises from coupling of incident light with the LSPR of individual nanosized metallic particles or with the near-field of nanogaps, nanotips, nanopores, and some of spatially confined sites, which can greatly enhance the Raman cross-section of the analyte adjacent to the site [12]. The highly concentrated EM field or the remarkably enhanced local optical field is widely acknowledged as a “hot spot”. Chemical enhancement is still under debate, but it is postulated to involve three distinct processes including (1) molecular excitation resonances; (2) charge transfer resonances; and (3) the static chemical enhancement [48,49]. Additionally, the CHEM enhancement is much weaker than the EM enhancement. Therefore, EM enhancement is a major concern in this study and the “hot spots” mainly occur at the nanogaps of the Au NPs for our obtained nanostructure. This type of substrate would provide an excellent SERS substrate for reliable and reproducible detection of analytes. 

**Figure 6:**
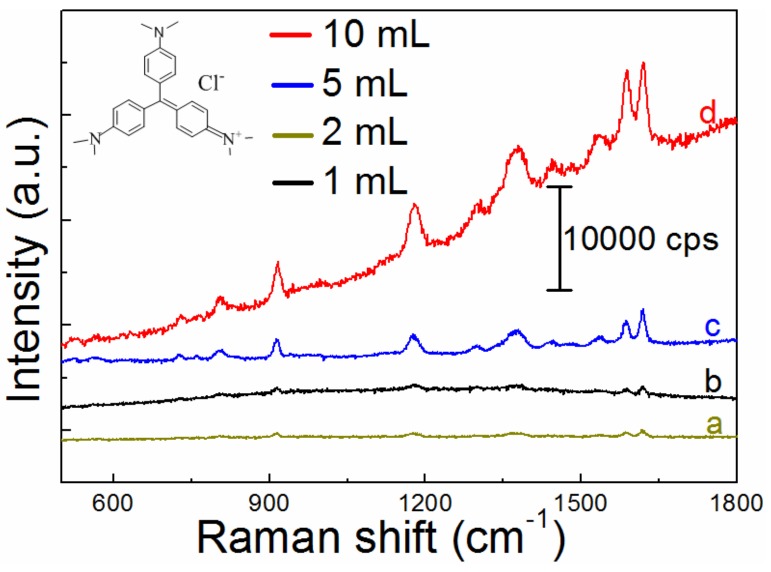
SERS enhancements of CVs (concentration: 10^−6^; soaking time: 15 min) on the prepared substrates activated by seed I and with adding different amounts of mixed solution of AA and Na_3_Cit with curves (**a**–**d**) corresponding to 1 mL, 2 mL, 5 mL, and 10 mL, respectively.

The following SERS measurements are focused on the seed I-activated substrates corresponding to the SEM images shown in Figure 3 and Figure 4. For example, Figure 6 showed the SERS spectra of CVs on substrates prepared by activation with seed I using different amounts of reaction solution. The SERS enhancement manifested a coverage dependence. The SERS enhancement of the substrate films increased with increasing the added reactant concentration. Strong SERS enhancements were achieved from the substrate film fabricated with over 5 mL of reactants (curves c and d in Figure 6). With increasing the film surface coverage and stacking density, there were more Raman molecules attaching to active sites for electromagnetic field enhancement. The characteristic vibrational bands of CVs were observed in all the SERS spectra. Intense Raman bands were located at around 1302 cm^−1^, 1445 cm^−1^, 1538 cm^−1^, 1589 cm^−1^, and 1620 cm^−1^, which were all related to the ring C–C stretching vibrations. Other peaks at 729 and 807 cm^−1^ were attributed to the C–H out-of-plane ring bending vibrations. The peak at 917 cm^−1^ was attributed to the ring skeletal vibrations of radial orientation. The broad peaks located at 1183 cm^−1^ and 1377 cm^−1^ were assigned to the C–H in-plane ring bending vibrations and the N-phenyl stretching vibrations, respectively. The breathing of central bonds, central C^+^-phenyl in-plane bending vibrations, and out-of-plane vibrations located at other low wave number position were very weak in all of Au NPs films [50,51]. Obviously, the main vibrational modes observed in SERS spectra on the prepared Au nanoparticles films were in good agreement with the reported results [50,51].

**Figure 7:**
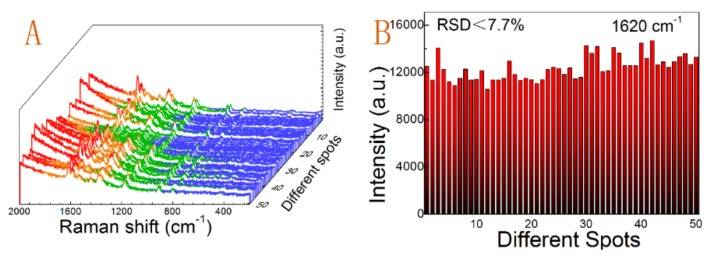
(**A**) Uniformity of SERS spectra of CVs collected on the randomly selected 50 spots of the whole substrate formed by seed I activation and 30 min of soaking, (**B**) the intensities of the main vibrations at 1620 cm^−1^ calculated according to the SERS spectra shown in (**A**).

The obtained substrates are very uniform with a flat surface and excellent light transmittance property under visual inspection, and the SEM images proved the uniformity of the substrate as mentioned above. We further checked the small spatial variation in SERS performance of the whole substrate. We selected the substrate activated with seed I and with 30 min of soaking time. Figure 7A showed the spectra of the SERS-active substrate with 50 randomly selected positions under identical experimental conditions. To illustrate and quantify the uniformity, we calculated the RSD values of the strongest peak at 1620 cm^−1^ in all SERS spectra given in Figure 7B. All prominent peaks of CV at 807 cm^−1^, 917 cm^−1^, 1183 cm^−1^, 1377 cm^−1^, 1589 cm^−1^, and 1620 cm^−1^ were listed in Table 2. All of the RSD values were far below 0.2, which suggested that the prepared substrates have excellent uniformity and reproducibility across the entire area of the substrate, according to the criterion propose by Tian et al. [52] 

Additionally, it is equally important to take an appropriate immersion time to fabricate large-area and uniform substrates. The uniformity of the SERS-active substrate formed by activation by seed I and 2 h of immersion was also used for the same analysis. Figure S3A–C exhibited SEM images and size distributions of the obtained Au nanoparticle films. All spectra of 60 random-spots were shown in Figure S3D. The values of prominent peaks at 1620 cm^−1^ were displayed in Figure S3E. Other RSD values of prominent peaks were listed in Table S1. However, we could clearly observe that the RSD values were greater. This was obviously due to the increased surface roughness of the substrate owing to physical adsorption of Au NPs and the existing defect regions. This phenomenon was consistent with the SEM results (Figure 3A1 and Figure S3A). Thus, the appropriate reactant concentration and immersion time for preparing large-area and highly uniform SERS substrate must always be considered. Thus, this study confirmed that the SERS-based substrate with great reproducibility, reliability, and uniformity is promising as an analysis platform for sensing applications.

## 5. Conclusions

In this work, we have fabricated a large-area and uniform SERS substrate on inexpensive and readily available polylysine-coated glass slide by seed-mediated electroless deposition (SMED) method. By employing this method, the large-area and uniform Au NPs films were directly deposited to the seed-modified glass substrate. Additionally, the activation procedure, the amount of reactant concentration, and the immersion time showed an important effect on the feature of the gold nanoparticle films. The substrate was prepared with seed I-activated and 30 min of soaking time, which exhibited an excellent uniformity and reproducibility in SERS measurements, with the RSD down to 8% (the minimum value was only 3% at 807 cm^−1^). This method can also be expandable to other polymer films, polymer modified or polymer-impregnated substrates. Furthermore, the large-area and uniform SERS substrates can serve as a promising candidate for applications in safety checks, optoelectronics, surface catalysis, etc.

## Figures and Tables

**Table 1:** Sample number and corresponding reaction conditions used to fabricate SERS-active substrates.

Sample	Seeds	Reactant Concentration	Soaking Time	Figure ID
1	-	10 mL	1 h	2(A,C)
2	-	10 mL	12 h	2(B,D)
3	Seed I	10 mL	30 min	3(A1–A4)
4	Seed II	10 mL	12 h	3(B1–B4)
5	Seed I	1 mL	12 h	4(A1–A3)
6	Seed I	2 mL	12 h	4(B1–B3)
7	Seed I	5 mL	12 h	4(C1–C3)
8	Seed II	5 mL	15 min	5(A1–A3)
9	Seed II	10 mL	30 min	5(B1–B3)
10	Seed II	10 mL	5 h	5(C1–C3)
11	Seed II	10 mL	20 h	5(D1–D3)
12	Seed I	10 mL	2 h	S3(A1–C3)

**Table 2:** RSD values for the major peaks of the SERS spectra for the substrate obtained with 30 min of immersion.

Peak Position (cm^−1^)	1620	1589	1377	1183	917	807
RSD values	0.077	0.069	0.059	0.047	0.036	0.031

## Supplementary Materials

The following are available online at http://www.mdpi.com/2079-4991/9/2/185/s1, Figure S1: UV-Vis spectra of Au seed solutions, Figure S2: UV-Vis spectra of Au nanoparticle films, Figure S3: SERS performance of Au nanoparticle films formed by seed I activation and with 2 h of immersion, Table S1: Relative standard deviation of the major peaks of the SERS spectra.
