# Supplementary material for: Seed-Mediated Electroless Deposition of Gold Nanoparticles for Highly Uniform and Efficient SERS Enhancement
Source: Nanomaterials (Basel). 2019 Feb 1;9(2):185. doi: 10.3390/nano9020185 (PMC6409782; doi:10.3390/nano9020185)
Supplement: Supplementary file 1 [file nanomaterials-09-00185-s001.pdf]

nanomaterials

Article

Seed-mediated electroless deposition of gold nanoparticles for highly uniform and efficient SERS enhancement

Junqi Tang [1,2], Quanhong Ou [1], Haichun Zhou [1], Limin Qi [2] and Shiqing Man [1,*]

1. UV-vis spectra of Au seed solutions

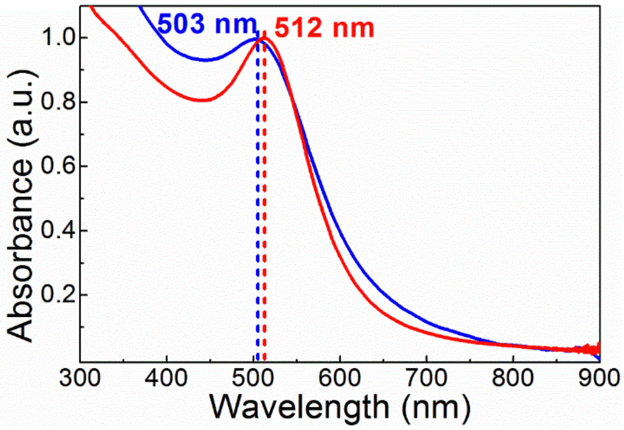

Figure S1. UV-vis spectra of Au seed solutions, blue curve) seed I, red curve) seed II. Both are normalized at the LSPR peak maximum.

2. UV-vis spectra of Au nanoparticle films

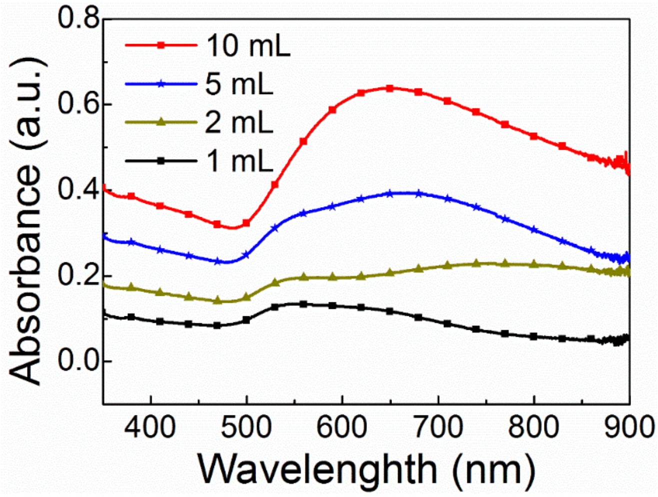

Figure S2. UV-vis spectra of the Au nanoparticle films formed by adding different amounts of reaction mixture after the substrates were activated with seed I.

3. SERS performance of Au nanoparticle films formed by seed I activation and with 2 h of immersion

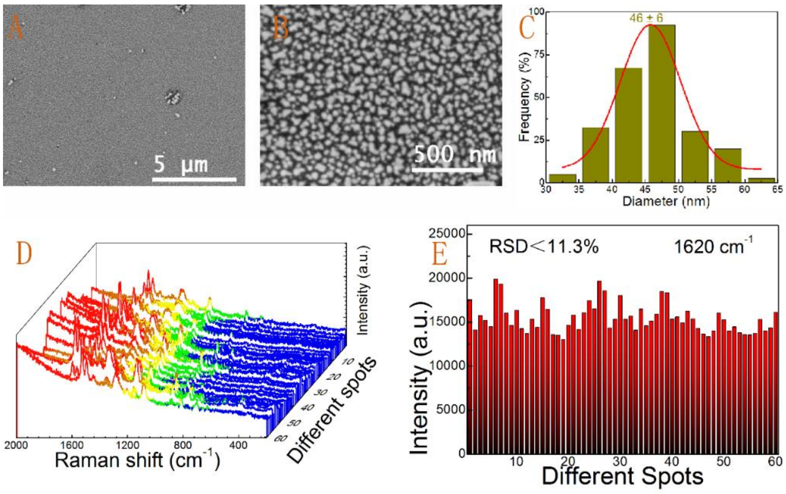

Figure S3. SEM images (A, B) and size distributions (C) of Au nanoparticle films formed by seed I activation and with 2 h. of immersion. D) Uniformity of SERS spectra of CVs collected on the randomly selected 60 spots of the whole substrate. E) The intensities of the main vibrations at 1620 cm^{-1} calculated according to the SERS spectra shown in (D).

4. Relative standard deviation of the major peaks of the SERS spectra

Table S1. RSD values for the major peaks of the SERS spectra for the substrate obtained with 2 h of immersion.

Peak Position (cm^{-1})	1620	1589	1377	1183	917	807
RSD values	0.113	0.095	0.085	0.077	0.067	0.056
